# UV-mediated thiol-ene click reactions for the synthesis of drug-loadable and degradable gels based on copoly(2-oxazoline)s

**DOI:** 10.1016/j.eurpolymj.2016.08.012

**Published:** 2016-08-20

**Authors:** Klaus P. Luef, Charlotte Petit, Bettina Ottersböck, Gernot Oreski, Francis Ehrenfeld, Bruno Grassl, Stéphanie Reynaud, Frank Wiesbrock

**Affiliations:** a Polymer Competence Center Leoben, Roseggerstrasse 12, 8700 Leoben, Austria; b Institute for Chemistry and Technology of Materials, Graz University of Technology, NAWI Graz, Stremayrgasse 9, 8010 Graz, Austria; c IPREM, UMR 5254 UPPA/CNRS, Hélioparc, 2 Avenue Du Président Angot, 64053 Pau CEDEX 09, France

**Keywords:** Poly(2-oxazoline), Crosslinked network, Glass-transition temperature, Hydrogel, Swelling degree, Thiol-ene click reaction

## Abstract

An 80-membered library of gels composed of monofunctional 2-ethyl-2-oxazoline and 2-nonyl-2-oxazoline and one of four selected difunctional 2-oxazolines (containing either ether or ester bonds) were synthesized by microwave-assisted ring-opening polymerizations. The difunctional 2-oxazolines were prepared from the thiol-ene reaction of glycol dimercaptoacetate or 2,2′-(ethylenedioxy)diethanethiol and 2-but-3′-enyl-2-oxazoline or 2-dec-9′-enyl-2-oxazoline. 53 of the gels exhibited glass-transition temperatures, which ranged from −5.9 to 45.3 °C. 13 Derivatives exhibited glass-transition temperatures in the range from 20 to 30 °C, which renders them stiff at room temperature and flexible at body temperature. The gels that did not contain any 2-ethyl-2-oxazoline acted as lipogels, whereas the gels that did not contain any 2-nonyl-2-oxazoline acted as hydrogels; all other gels may be classified as amphigels. The swelling degrees were measured by gravimetry and maximum swelling degrees of 6 (in water) were observed for the gels with the lowest degrees of crosslinking. In a second approach, the synthesis of crosslinked networks had been achieved by performing the polymeranalogous thiol-ene reaction of copoly(2-oxazoline)s containing olefinic side-chains and glycol dimercaptoacetate. This soft strategy enabled the straightforward loading of such gels with active pharmaceutical ingredients without altering them. This method delivered gels with selected composition exhibiting a targeted disc-shape and loaded with active pharmaceutical ingredients from one-step syntheses. The maximum swelling degrees of these specimens were found to be in accordance with the ones from the first route investigated. Preliminary degradation studies were performed at 25 °C; these types of gels were found to be degraded in alkaline media as well as by esterases.

## 1 Introduction

**Scheme 1:**
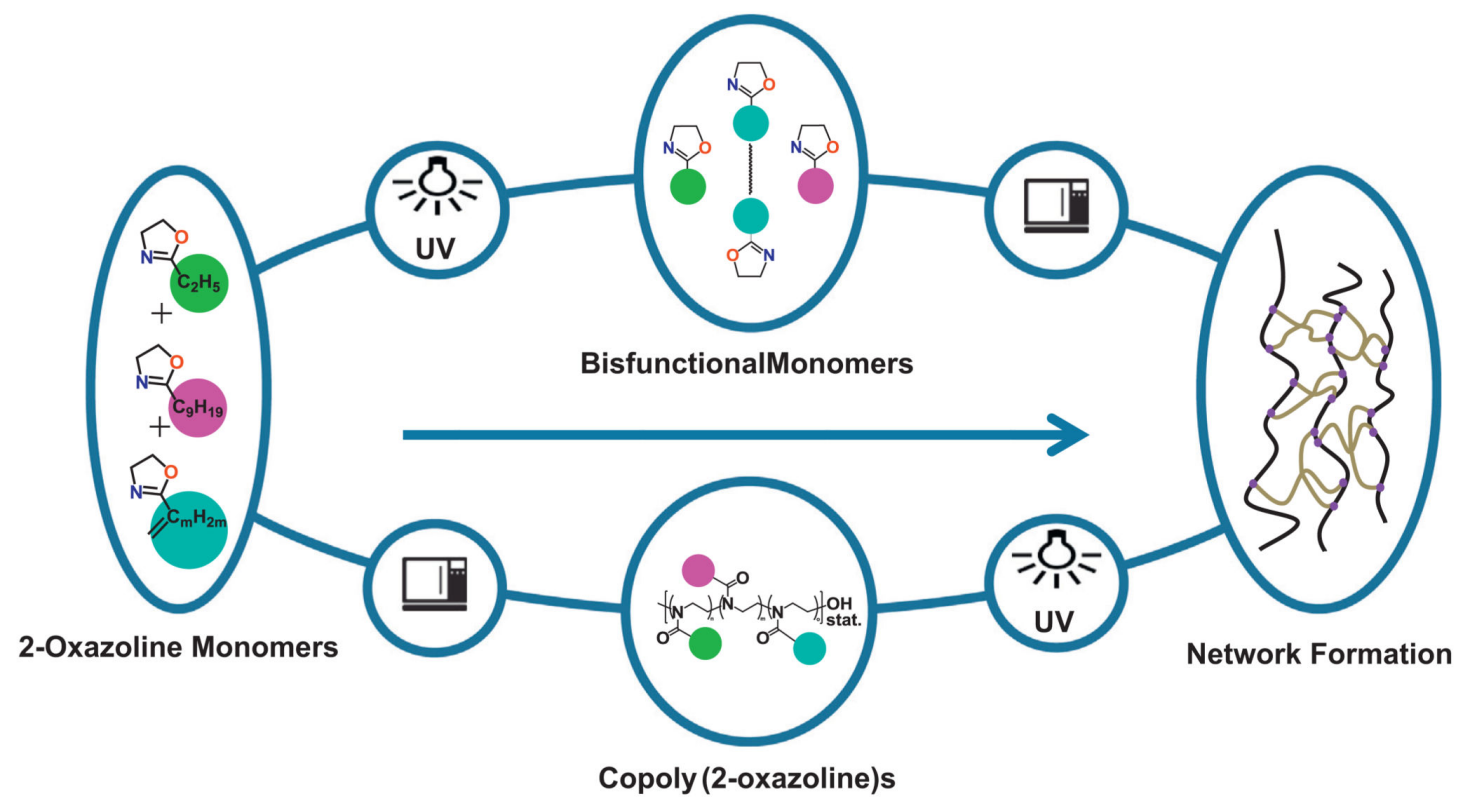
Route 1 (top) and Route 2 (bottom) for the synthesis of crosslinked networks investigated in this study. 2-But-3′-enyl-2-oxazoline (m = 3) and 2-Dec-9′-enyl-2-oxazoline (m = 9) were used as olefinic monomers.

No other polymer class has benefited from the advent of microwave reactors in chemical laboratories like the poly(2-oxazoline)s. These polymers and corresponding copolymers can be synthesized by the cationic ring-opening polymerization of 2-oxazoline monomers (Scheme 1), which inherently bears the advantage to introduce a variety of functionalities in their side-chains [1–4]. The structural motif of 2-oxazolines is the five-membered heterocycle containing an oxygen atom and a nitrogen atom (and one double bond). The initiation of the polymerization occurs regioselectively at the nitrogen atom [5,6], yielding polymers with a side-chain amide bond per repetition unit (Scheme 1), classifying poly(2-oxazoline)s as so-called pseudo-peptides, which renders them potential candidates for medical and medicinal applications.

Nonetheless, due to their low polymerization rates (as compared to other polymer classes), poly(2-oxazoline)s have been hibernating for the last two decades of the precedent millennium, after their discovery in 1966/67 [7–10], and intense research for almost two decades. They benefit from the abovementioned microwave reactors as those offer easy access to autoclave conditions and, consequently, facilitate overcoming low polymerization rates by eliminating the temperature limitation by boiling points [11,12]. Consequently, research for these polymers has been performed in intense manner again.

One direction of research efforts currently investigates the application of poly(2-oxazoline)s for drug delivery purposes and tissue engineering [13–15]. Crosslinked poly(2-oxazoline)s [16,17] have been studied in this context for cellular uptake [18], tissue engineering [19], and preferred adhesion to cancer cells [20]. The variety of potential applications elucidates the long lists of demands for gels in this field of applications.

In this context, the first part of the present study has been performed to correlate thermal transitions and swelling properties with the molecular structure of the networks constituting lipo-, amphi- and hydrogels in order to determine general trends of a library of 80 gels made from poly(2-oxazoline)-based networks (Scheme 1: Route 1). The second part has been dedicated to the applicability of such polymer networks for medic(in)al applications. For that approach, disc-shaped gels having a composition selected among the abovementioned 80 gels library were produced with or without active pharmaceutical ingredient (API) (Scheme 1: Route 2). In addition, an optometrical swelling degree determination has been performed and compared to the gravimetric one.

**Scheme 2:**
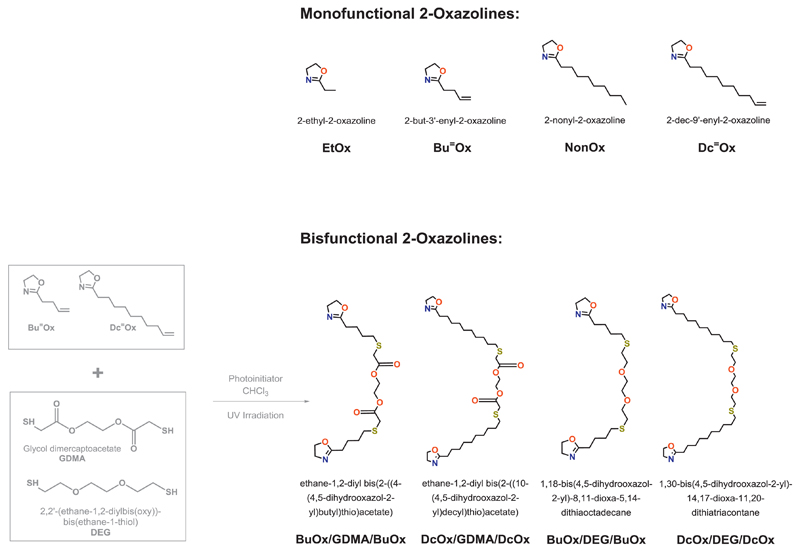
Structures of the 2-oxazoline monomers investigated in this study.

Networks with hydrolysable ester bonds and non-hydrolysable ether bonds, in addition to the amide bonds of the poly(2-oxazoline)s themselves, were considered in both parts of this study (Scheme 2); first degradation studies were performed with one gel that contained ester bonds in the pH range from 4 to 10 without and with esterases.

It is worth mentioning that the glass-transition temperatures are one key criterion for the decision if crosslinked polymers may be successfully implanted into mammalian bodies: While the polymers should be sufficiently stiff during the implanting surgery, they should be above their glass-transition temperature after they have reached ‘body temperature’ (their application temperature) in order not to be brittle – this prerequisite is indicative of favorable glass-transition temperatures between 20 and 30 °C. Notably, the uptake of water by the gels, in particular by hydrogels, under physiological conditions additionally lowers the glass-transition temperature of the swollen gel compared to the gel in its dry state [21]. The swelling behavior of the gels in model solvents, namely water, ethanol, and dichloromethane, was investigated, on the one hand enabling their classification as hydro-, amphi-, or lipogel. The swelling properties were determined for all gels in the powdered form (Scheme 1, Route 1) and, for selective examples, cross-checked for the disc form (Scheme 1, Route 2).

## 2 Experimental

### 2.1 Materials

2-Ethyl-2-oxazoline (EtOx) and methyl tosylate (MeOTs) were purchased from Sigma-Aldrich (Vienna, Austria), purified by distillation over sodium sulfate and stored in inert atmosphere. 2,2′-(Ethylenedioxy)diethanethiol (DEG), dichloromethane, ethanol and deuterated chloroform were acquired from Sigma-Aldrich (Vienna, Austria) and used without purification. Glycol dimercaptoacetate (GDMA) was kindly provided by Bruno Brock (Marschacht, Germany) and used without any further purification. The photoinitiator Lucirin TPO-L^®^, ethyl-2,4,6-trimethylbenzoylphenylphosphinate, was purchased from abcr GmbH (Karlsruhe, Germany). Deionized water was produced through reverse osmosis. 2-But-3′-enyl-2-oxazoline (Bu⁼Ox), 2-nonyl-2-oxazoline (NonOx) and 2-dec-9′-enyl-2-oxazoline (Dc⁼Ox) were synthesized according to literature protocols [22–24], purified by distillation and flash chromatography using chloroform as an eluent. For the degradation studies, the enzymes (namely porcine liver esterase and rabbit liver esterase), Eosin B, and the buffer solutions (pH = 4, 6, 8, and 10) were acquired from Sigma-Aldrich (Vienna, Austria) and used as received.

### 2.2 Instrumentation

Syntheses were performed in the microwave reactor Initiator 2.5 from Biotage in sealed vials. All syntheses were performed in temperature-controlled modes; the reaction temperature of 140 °C was monitored by a non-invasive IR pyrometer, the calibration of which was controlled regularly. For the small-scale syntheses described in this study, input powers of fewer than 60 W were sufficient to maintain the reaction temperatures. ^1^H NMR spectra were measured in deuterated chloroform on a Bruker 300 MHz spectrometer. The residual solvent peaks were used for referencing the spectra. UV-irradiation was realized with a Hg/Xe lamp of the EFOS Novacure from EXFO. The thermal analyses were performed on thoroughly dried samples using a Perkin Elmer DSC 4000 machine (Waltham, USA). Indium and Zinc standards were used for the temperature and enthalpy calibration. UV–vis spectra were measured with the Shimadzu UV–vis spectrophotometer 1800. Samples of approximately 10 mg were placed into 50 μL pans with perforated lids. Thermograms were recorded under a nitrogen atmosphere from −50 to 100 °C. A heating rate of 20 K min^−1^ was chosen for the determination of the glass-transition temperatures. Two measurements were performed per sample after an initial first heating run that was not considered for the subsequent calculations. The optometric monitoring of the swelling properties of gels were recorded on a home-made device called FDRRT (Find Disc Radius in Real Time), an improved version of the SKM system [25]. During measurement, the gels were lighted up with a LED lamp, ensuring no heat transfer to the solvent and an enhanced contrast between the gel and solvent for video detection. The swelling was monitored using a CCD camcorder, 15 fps (Sony XC-75, 1024 × 768) for horizontal detection and a video CCD camcorder, 30 fps (SVS-VISTEK GigE, 1280 × 960), for vertical detection, both connected to a PC via a labview software.

### 2.3 Synthesis of difunctional 2-oxazolines

The synthesis of the difunctional 2-oxazoline monomers was accomplished by UV-mediated thiol-ene click reactions in quantitative yield, involving one equivalent of a dithiol compound, namely DEG or GDMA, and 2 equivalents of a 2-oxazoline monomer with an olefinic side-chain, namely Bu⁼Ox or Dc⁼Ox. From these reactions, four difunctional 2-oxazoline monomers were synthesized: BuOx/DEG/BuOx, DcOx/DEG/DcOx, BuOx/GDMA/Buox, and DcOx/GDMA/DcOx. In a typical experiment, 6.80 g (32.4 mmol, 2 eq) of Dc⁼Ox were dissolved in 25 mL of chloroform. To this solution, 3.59 g of GDMA (17 mmol, 1.05 eq) and 250 mg of photoinitiator Lucirin TPO-L were added, and the reaction mixture was irradiated at 4.5 W cm^−2^ under constant stirring for 0.5 h. After illumination, the solvent was evaporated under reduced pressure, quantitatively yielding the product DcOx-GDMA-DcOx, which was stored at 4 °C.

#### 2.3.1 Analytical data of BuOx/GDMA/BuOx

^1^H NMR (300 MHz, CDCl_3_): *δ* (ppm) = 1.50–1.88 (8 H, m, 4 —*CH_2_*—), 2.19–2.49 (4 H, m, 2 =C(−)—*CH_2_*—CH_2_—), 2.66 (4 H, t, ^3^*J*_H—H_ = 7.2 Hz, 2 —CH_2_—*CH_2_*—S—), 3.24 (4 H, s, 2 —S—*CH_2_*—C(=O)—), 3.82 (4 H, t, ^3^*J*_H—H_ = 9.3 Hz, 2 —C=O—*CH_2_*—CH_2_—N=), 4.23 (4 H, t, ^3^*J*_H—H_ = 9.3 Hz, 2 —C=O—CH_2_—*CH_2_*—N=), 4.30–4.45 (4 H, m, 2 —O—*CH_2_*—CH_2_—).

^13^C NMR (300 MHz, CDCl_3_): *δ* (ppm) = 25.0, 27.4, 28.4, 32.2, 33.4, 54.3, 62.7, 67.2.

IR (ATR, cm^−1^): ν = 418, 522, 550, 665, 748, 852, 952, 1044, 1122, 1263, 1438, 1662, 1732, 2940, 3291.

#### 2.3.2 Analytical data of BuOx/DEG/BuOx

^1^H NMR (300 MHz, CDCl_3_): *δ* (ppm) = 1.53–1.82 (8 H, m, 4 —*CH_2_*—), 2.19–2.44 (4 H, m, 2 =C(−)—*CH_2_*—CH_2_—), 2.56 (4 H, t, ^3^*J*_H—H_ = 6.1 Hz, 2 —CH_2_—*CH_2_*—S—), 2.70 (4 H, t, ^3^*J*_H—H_ = 6.7 Hz, 2 AS—*CH_2_*—CH_2_—), 3.48–3.73 (8 H, m, 4 —O—*CH_2_*—CH_2_—), 3.81 (4 H, t, ^3^*J*_H—H_ = 9.3 Hz, 2 —C=O—*CH_2_*—CH_2_—N=), 4.22 (4 H, t, ^3^*J*_H—H_ = 9.3 Hz, 2 —C=O—CH_2_—*CH_2_*—N=).

^13^C NMR (300 MHz, CDCl_3_): *δ* (ppm) = 25.1, 27.5, 29.2, 31.4, 32.1, 54.3, 67.2, 70.3, 71.0.

IR (ATR, cm^−1^): ν = 550, 750, 912, 952, 985, 1042, 1103, 1170, 1218, 1291, 1352, 1436, 1664, 1732, 2911.

#### 2.3.3 Analytical data of DcOx/GDMA/DcOx

^1^H NMR (300 MHz, CDCl_3_): *δ* (ppm) = 1.19–1.44 (24 H, m, 12 —*CH_2_*—), 1.50–1.69 (8 H, m, 4 —*CH_2_*—), 2.25 (4 H, t, ^3^*J*_H—H_ = 7.4 Hz, 2 =C(−)—CH_2_—CH_2_—), 2.62 (4 H, t, ^3^*J*_H—H_ = 7.2 Hz, 2 —CH_2_—*CH_2_*—S—), 3.22 (4 H, s, 2 —S—*CH_2_*—C(=O)—), 3.81 (4 H, t, ^3^*J*_H—H_ = 9.3 Hz, 2 —C=O—*CH_2_*—CH_2_—N=), 4.21 (4 H, t, ^3^*J*_H—H_ = 9.3 Hz, 2 —C=O—CH_2_—*CH_2_*—N=), 4.35 (4 H, s, 2 —O—*CH_2_*—CH_2_—).

^13^C NMR (300 MHz, CDCl_3_): *δ* (ppm) = 25.9, 28.0, 28.7, 28.9, 29.2, 29.2, 29.4, 29.4, 32.7, 33.4, 54.3, 62.7, 67.1.

IR (ATR, cm^−1^): ν = 424, 554, 699, 750, 954, 1042, 1171, 1211, 1293, 1379, 1554, 1664, 1734, 2850, 2919.

#### 2.3.4 Analytical data of DcOx/DEG/DcOx

^1^H NMR (300 MHz, CDCl_3_): *δ* (ppm) = 1.17–1.44 (24 H, m, 12 —*CH_2_*—), 1.48–1.70 (8 H, m, 4 —*CH_2_*—), 2.26 (4 H, t, ^3^*J*_H—H_ = 6.7 Hz, 2 =C(−)—*CH_2_*—CH_2_—), 2.53 (4 H, t, ^3^*J*_H—H_ = 7.2 Hz, 2 —CH_2_—*CH_2_*—S—), 2.70 (4 H, t, ^3^*J*_H—H_ = 7.0 Hz, 2 —S—*CH_2_*—CH_2_—), 3.54–3.70 (8 H, m, 4 —O—*CH_2_*—CH_2_—), 3.81 (4 H, t, ^3^*J*_H—H_ = 9.3 Hz, 2 —C=O—*CH_2_*—CH_2_—N=), 4.22 (4 H, t, ^3^*J*_H—H_ = 9.3 Hz, 2 —C=O—CH_2_—*CH_2_*—N=).

^13^C NMR (300 MHz, CDCl_3_): *δ* (ppm) = 25.9, 27.9, 28.8, 29.2, 29.4, 29.4, 29.8, 31.4, 32.6, 54.2, 67.2, 70.3, 71.0.

IR (ATR, cm^−1^): ν = 501, 550, 663, 750, 852, 914, 985, 1042, 1128, 1216, 1464, 1664, 1734, 2849, 2917.

### 2.4 In-situ preparation of 2-oxazoline hydrogels

Hydrogels were prepared from the copolymerization of mono- and difunctional 2-oxazoline monomers by microwave-assisted cationic ring-opening polymerizations. EtOx and NonOx were chosen as monofunctional 2-oxazoline monomers, and BuOx/DEG/BuOx, DcOx/DEG/DcOx, BuOx/GDMA/Buox, and DcOx/GDMA/DcOx were reacted as difunctional 2-oxazolines. MeOTs was applied as initiator, and dry chloroform was used as the solvent. The ratios of the monofunctional 2-oxazoline monomers were chosen from [EtOx]:[NonOx] = 150:0, 100:50, 50:100, or 0:150 (4 ratios), the ratio [MeOTs]: [EtOx + NonOx] was kept constant at 1:150, and the ratio of monofunctional:difunctional 2-oxazoline monomers was varied according to 150:30, 150:15, 150:10, 150:7.5, 150:6, (5 ratios), yielding 4 libraries (one for each difunctional monomer) of 20 members each, respectively 80 gels in total. In a typical procedure, a ratio of [EtOx]:[NonOx] of 100:50, a ratio of [EtOx + NonOx]:[DcOx/DEG/DcOx] of 150:6, and a ratio of [EtOx + NonOx]:[MeOTs] of 150:1 was mixed from 0.763 g (7.69 mmol, 100 eq) EtOx, 0.759 g (3.845 mmol, 50 eq) NonOx, 0.278 g (0.4614 mmol, 6 eq) DcOx/DEG/DcOx, and 14.3 mg (0.0769 mmol, 1 eq) MeOTs. The mixture was placed in a microwave vial under inert gas (argon) and was sealed with a septum. Microwave irradiation was applied at 140 °C for 1 h. The colorless to slightly yellow solid products were recovered and subjected to swelling/drying cycles in dichloromethane until weight constant was reached. The yields of the purified gels were over 93%.

### 2.5 Polymeranalogous preparation of 2-oxazoline hydrogels

The polymeranalogous strategy aimed for the reproduction of gels with the same composition like the gels that were produced in-situ (Section 2.4). Hence, linear copoly(2-oxazoline)s composed of EtOx, NonOx and either Bu⁼Ox or Dc⁼Ox were prepared by microwave-assisted cationic ring-opening polymerizations. These copolymers with olefinic side-chains of chosen compositions were crosslinked with a dithiol compound, namely either DEG or GDMA, by thiol-ene click reaction in a second step. In a typical experiment, 2.00 g of the copolymer pEtOx_150_-*stat*-pBu⁼Ox_30_ (containing 3.22 mmol or 2 equivalents of Bu⁼Ox) was dissolved in 3 mL of chloroform and mixed with 0.339 g of GDMA as crosslinker (correlating to 1.61 mmol or 1 equivalent) and 100 mg of photoinitiator Lucirin TPO-L. Eventually, 100 mg ibuprofen (for the study aiming at the optometric determination of swelling degrees) were added to the mixture. The reaction mixture was poured into a steel template for presetting the geometry of discs of 50 mm diameter and 2 mm height and UV-irradiated with an intensity of 5.5 W cm^−2^ for 15 min.

### 2.6 Determination of swelling degrees

For the gravimetric determination of swelling degrees, powder samples (0.1 g) of each of the 80 gels were swollen in excess amounts of the respective solvents (water, ethanol, and dichloromethane). The swelling degrees were determined gravimetrically after 24 and 48 h after storing the swollen gels on cellulose-based tissue paper until solvent was no longer released. For the optometric detection of swelling degrees, polymer discs with a diameter of 3 mm were punched out from the 50 mm discs and dried until weight constancy. Gel films were then placed in a FDRRT cell (lead-free optical glass) and immersed in an excess of solvent. All the experiments were performed at room temperature without stirring. FDRRT gives the measurement of the gel characteristics in precise and time-efficient manners avoiding all the tedious and cumbersome steps of the weighting techniques. As compared to the previous SKM development [25], FDRRT is appropriate for cylinder and also disc and spherical gels and determines the gel size more accurately thanks to the up-graded camcorders and software.

### 2.7 Degradation studies

Degradation studies were performed on discs of pEtOx_100_-*stat*-pNonOx_50_-*stat*-pBu⁼Ox_30_ crosslinked with GDMA. In a typical procedure, 2.00 g of the copolymer pEtOx_100_-*stat*-pNonOx_50_-*stat*-pBu⁼Ox_30_ (containing 3.22 mmol or 2 equivalents of Bu⁼Ox) was dissolved in 3 mL of chloroform and mixed with 0.339 g of GDMA as crosslinker (correlating to 1.61 mmol or 1 equivalent), 8.0 mg of Eosin B, and 100 mg of photoinitiator Lucirin TPO-L. The reaction mixture was poured into a steel template for presetting the geometry of discs of 50 mm diameter and UV-irradiated with an intensity of 5.5 W cm^−2^ for 15 min. From these large discs, small discs with a diameter of 2 mm were cut. The degradation studies were performed by storing sets of four small discs in 2.5 mL of an aqueous solution at pH = 4, 6, 8, and 10 without additional enzymes, as well as in 2.5 mL of an aqueous solution at pH = 8 with either porcine liver esterase (PLE) or rabbit liver esterase (RLE) at 25 °C. The esterases were added in quantities of 200 units per 2.5 mL. The degradation was quantified by the release of the dye Eosin B, which was detected by UV/Vis spectrometry. From these measurements, the quantity of Eosin B was calculated from the difference of the absorbances at 515 and 800 nm.

## 3 Results and discussion

### 3.1 Library planning

In order to cover a broad spectrum of material properties regarding hydrophobicity and thermal behavior with special respect to the different prerequisites in individual medic(in)al applications, six different 2-oxazoline-based monomers were chosen for the synthesis of four 20-membered libraries of networks (Scheme 2). All polymer networks were composed of monofunctional EtOx and/or NonOx (four different ratios with respect to the initiator, namely 150:0, 100:50, 50:100, and 0:150) and one of the four difunctional 2-oxazoline monomers (Scheme 2; five different ratios with respect to the equivalents of EtOx and NonOx = 150, namely 150:30, 150:15, 150:10, 150:7.5, and 150:6).

The commercially available EtOx was the preferred hydrophilic component, whereas NonOx was chosen as its hydrophobic counterpart due to its simple and eco-friendly synthesis by the reaction of decanoic acid from coconut oil with ethanol amine. Both monomers can be statistically polymerized. The four different difunctional 2-oxazolines were synthesized from two unsaturated 2-oxazoline monomers, namely Bu⁼Ox and Dc⁼Ox, and two dithiol crosslinkers, namely DEG and GDMA. The hydrophilic Bu⁼Ox monomer with its short non-conjugated unsaturated side-chain was synthesized according to literature protocols in a solvent-extensive 3-step procedure [21], whereas the hydrophobic Dc⁼Ox was synthesized in analogy to NonOx in a solvent-free, one-step synthesis [26] from renewable resources (involving undecenoic acid from castor oil).

*Route 1* (Scheme 1): From Bu⁼Ox and Dc⁼Ox, respectively, difunctional 2-oxazoline monomers were obtained by the UV-mediated thiol-ene click reaction with two different commercially available dithiols, namely GDMA and DEG, yielding the four different difunctional oxazolines, BuOx/DEG/BuOx, DcOx/DEG/DcOx, BuOx/GDMA/BuOx, and DcOx/GDMA/DcOx (Scheme 1). GDMA was chosen because of its ester functionalities, making it an ideal target for acidic/alkaline and/or enzymatic degradation, paving the way for stimuli-induced compound release of molecules occluded in the polymer network. DEG represents the structure-analogous dithiol with two ether functionalities, rendering network degradation hardly feasible.

Following a microwave-assisted in-situ polymerization strategy, varying ratios of monofunctional EtOx and NonOx as well as one of the difunctional 2-oxazoline crosslinkers were copolymerized, resulting in a 4 × 20 = 80-membered network library. After copolymerization, the hydrogels were purified by repeating cycles of swelling-recovery-drying in dichloromethane, with yields of 93% and higher. Purified gels had a colorless to slight yellow appearance and showed brittle behavior in their dried state.

*Route 2* (Scheme 1): Notably, for the production of gels of defined shapes, if monofunctional EtOx and NonOx were copolymerized with either Bu⁼Ox or Dc⁼Ox, crosslinking of the thus-obtained linear copoly(2-oxazoline)s could also be accomplished by subsequent polymeranalogous UV-mediated thiol-ene reactions with either one of the difunctional dithiol compounds, GDMA or DEG (see Section 3.4). By preparing a solution of the copolymer of the composition poly(EtOx)_n_-*stat*-poly(NonOx)_n_-*stat*-poly(X)_n_ (X = Bu⁼Ox or Dc⁼Ox), the dithiol GDMA or DEG, and the photoinitiator Lucirin TPO-L in the solvent chloroform prior to the UV-induced crosslinking, the shape of the resulting polymer network could be defined by the form of the template in which the formulation was poured. With this method, polymer discs of 50 mm diameter and 1 mm height could be obtained. Following the analogous purification route as for the in-situ produced networks, discs of the pre-set/targeted dimensions were obtained.

### 3.2 Thermal transitions

With special respect to the narrow frame for the targeted glass-transition temperatures of above 20 °C and below 30 °C, it is important to consider the most common repetition units of the networks, namely pEtOx and pNonOx. While no glass-transition temperature has been reported for pNonOx in the range to temperatures as low as −150 °C, the glass-transition temperature for pEtOx has been reported as 59.3 ± 1.6 [27], 61 [28], and 70 °C [29], respectively. Hence, at an initial stage of this study, the glass-transition temperatures of the linear copoly(2-oxazoline)s composed of pEtOx and pNonOx were determined as follows: 56.4 ± 0.2 °C (pEtOx_150_), 20.0 ± 0.1 °C (pEtOx_100_-*stat*-pNonOx_50_), and 16.5 ± 0.1 °C (pEtOx_50_-*stat*-pNonOx_100_); no glass-transition temperature was found for pNonOx_150_ either. These data convey the general concept that an increasing amount of pNonOx in the crosslinked networks (to the disadvantage of the content of pEtOx), consequently lowers the glass-transition temperature of the network.

**Fig. 1:**
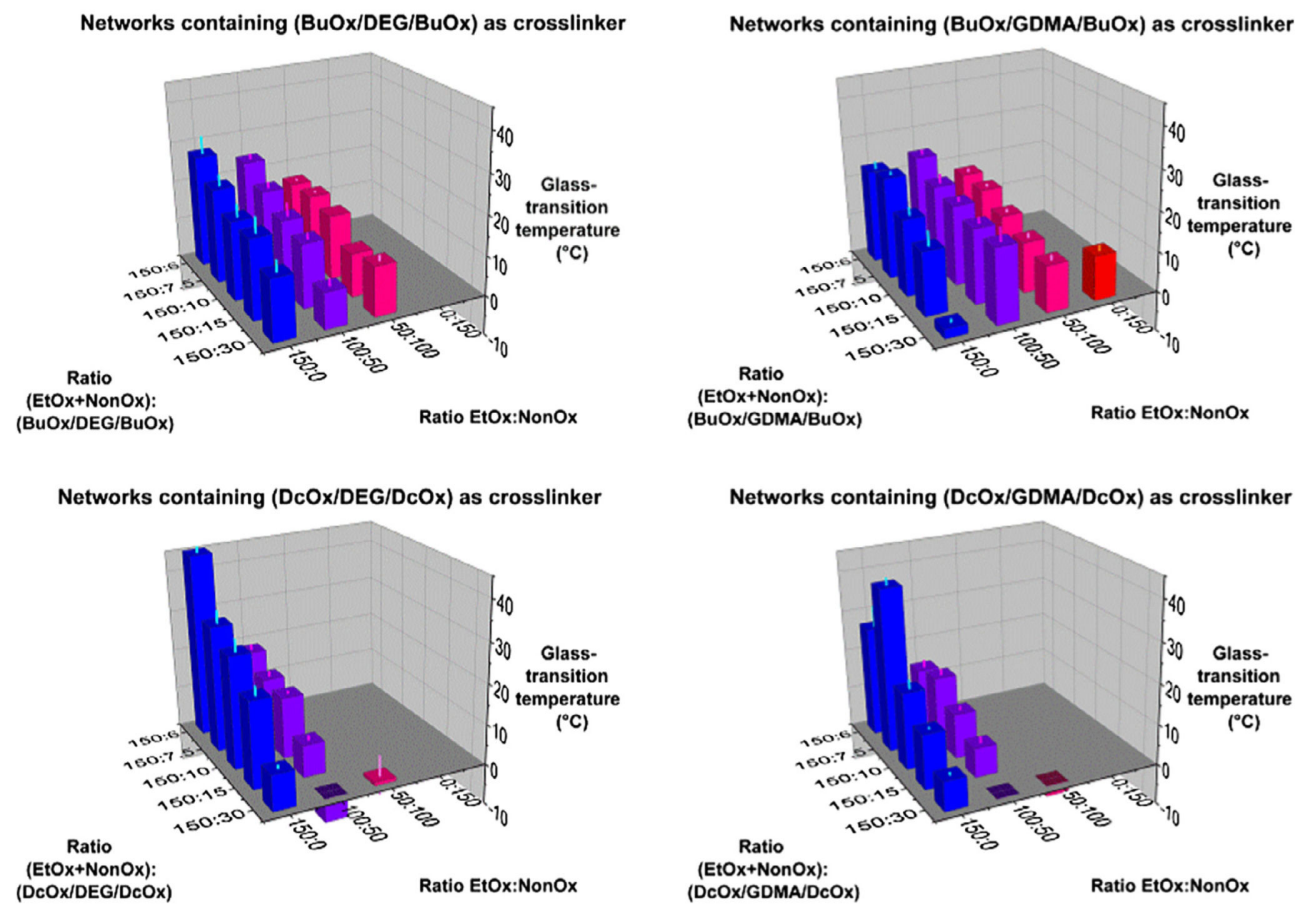
Glass-transition temperatures of the copoly(2-oxazoline)-based networks.

The thermal analysis by differential-scanning calorimetry of the 80 networks in their dry state revealed that none of the compounds exhibited a melting point. Regarding the glass-transition temperatures of the networks (Fig. 1; see also Table SI-1 in the Supporting Information), four general trends can be easily recognized: (i) Of the crosslinked polymers that did not contain any pEtOx, only one congener (pNonOx_150_-*stat*-p(BuOx/GDMA/BuOx_30_) showed a glass-transition of 11.1 ± 1.2 °C in the measured range of temperatures. (ii) For the pEtOx_50_-*stat*-pNonOx_100_ series, the longer the chain length of the difunctional 2-oxazoline monomer was (BuOx/X/BuOx vs. DcOx/x/DcOx; X = DEG or GDMA), the lower the glass-transition temperature was. This phenomenon can be recognized in particular by the fact that the networks containing DcOx/X/DcOx (X = DEG or GDMA) as crosslinker, while only the compounds with the highest amount of crosslinker investigated in this study showed any glass-transition at all (pEtOx_50_-*stat*-pNonOx_100_-*stat*-p(DcOx/DEG/DcOx)_30_: 0.8 ± 4.9 °C; pEtOx_50_-*stat*-pNonOx_100_-*stat*-p(DcOx/GDMA/DcOx)_30_: -2.6 ± 0.2 °C). (iii) All 40 congeners derived from the pEtOx_150_ and pEtOx_100_-*stat*-pNonOx_50_ series showed a glass-transition. (iv) In the the pEtOx_150_ and pEtOx_100_-*stat*-pNonOx_50_ series, an increasing amount of crosslinker in the range from 150:6 to 150:30 lowers the respective glass-transition temperature of the network.

Considering the networks investigated, it may be summarized that 53 of the 80 crosslinked polymers exhibited a glass-transition temperature within the investigated range of temperatures. The glass-transition temperatures spanned a range from −5.9 to 45.3 °C; a total of 13 derivatives exhibited glass-transition temperatures in the targeted range of 20–30 °C. Notably, each of the four sub-libraries of this study contained at least one congener that met the targeted temperature window.

### 3.3 Gravimetric determination of swelling degrees (Route 1 of Scheme 1)

**Fig. 2:**
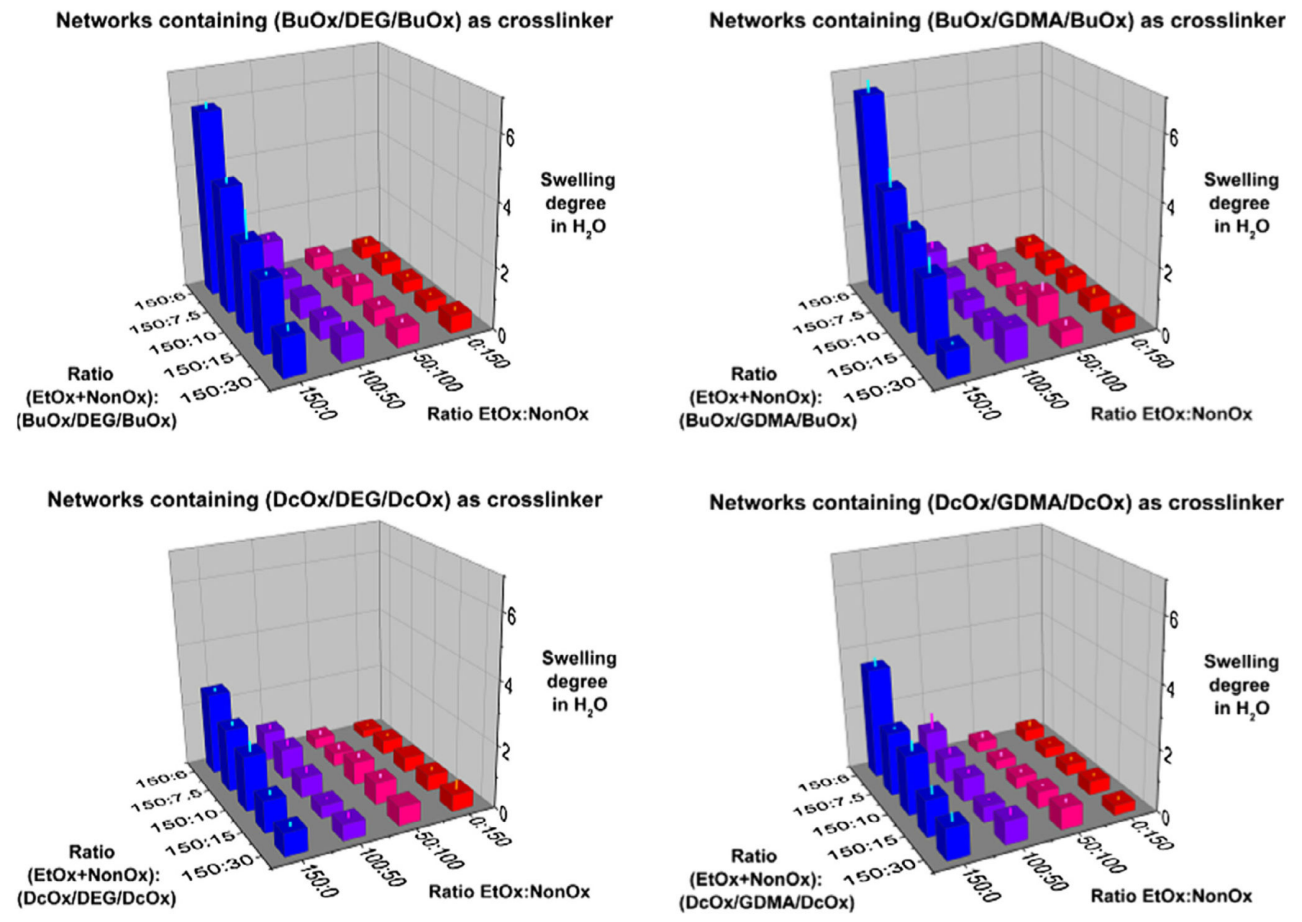
Swelling degrees of the copoly(2-oxazoline)-based networks in water.

**Fig. 3:**
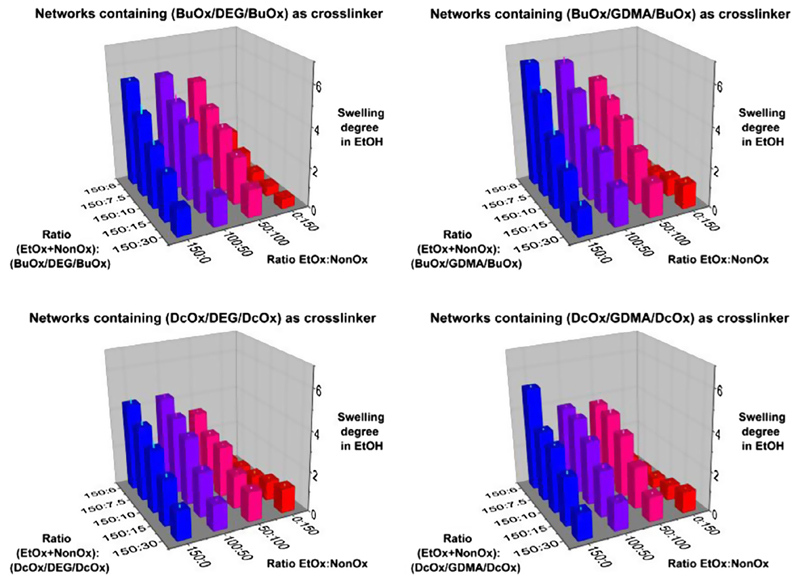
Swelling degrees of the copoly(2-oxazoline)-based networks in ethanol.

**Fig. 4:**
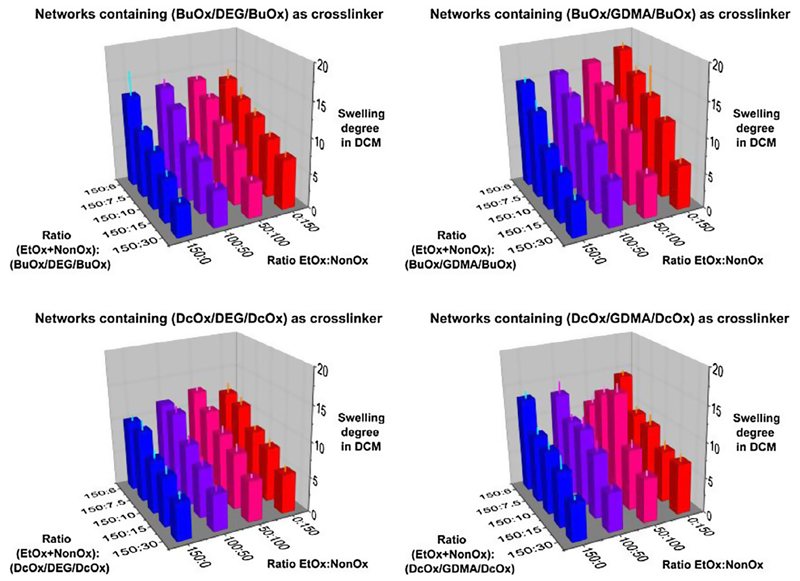
Swelling degrees of the copoly(2-oxazoline)-based networks in dichloromethane.

The swelling degrees were determined in three representative test solvents: water, ethanol and dichloromethane (Figs. 2–4). The three test solvents were chosen due to the pronounced hydrophilicity of pEtOx (that is water-soluble), aiming at a clear distinction among the pEtOx_150_, pEtOx_100_-*stat*-pNonOx_50_, pEtOx_50_-*stat*-pNonOx_100_, and pNonOx_150_ series. Maximum swelling was determined within 48 h by swelling a powdered gel sample in the respective solvent, careful removal of any excess solvent and subsequent gravimetrical analysis.

Swelling of the gels in water (Fig. 2; see also Table SI-2 in the Supporting Information) to significant extent could only be observed for the gels that did not contain any pNonOx repetition units. Hence, only the gels of the composition pEtOx_150_-*stat*-p(BuOx/DEG/BuOx)_n_, pEtOx_150_-*stat*-p(BuOx/GDMA/BuOx)_n_, pEtOx_150_-*stat*-p(DcOx/DEG/DcOx)_n_, and pEtOx_150_-*stat*-p(DcOx/GDMA/DcOx)_n_ (20 members) may be classified as hydrogels. In these four series of gels, an increasing degree of crosslinking, represented by the content of difunctional 2-oxazolines, increased the network rigidity and lowered the maximum swelling degrees. A maximum swelling degree in the range of 6 was observed for the gels pEtOx_150_-*stat*-p(BuOx/DEG/BuOx)_6_ and pEtOx_150_-*stat*-p(BuOx/GDMA/BuOx)_6_. The swelling degrees of the gels that contained Dc⁼Ox-derived crosslinkers were lower than those of the gels that contained Bu⁼Ox-derived crosslinkers.

All gels that contained repetition units of pEtOx showed swelling properties in ethanol (Fig. 3; see also Table SI-3 in the Supporting Information). Hence the gels from eight series, namely pEtOx_100_-*stat*-pNonOx_50_-*stat*-p(BuOx/DEG/BuOx)_n_, pEtOx_50_-*stat*-pNonOx_100_-*stat*-p(BuOx/DEG/BuOx)_n_, pEtOx_100_-*stat*-pNonOx_50_-*stat*-p(BuOx/GDMA/BuOx)_n_, pEtOx_50_-*stat*-pNonOx_100_-*stat*-p(BuOx/GDMA/BuOx)_n_, pEtOx_100_-*stat*-pNonOx_50_-*stat*-p(DcOx/DEG/DcOx)_n_, pEtOx_50_-*stat*-pNonOx_100_-*stat*-p (DcOx/DEG/DcOx)_n_, pEtOx_100_-*stat*-pNonOx_50_-*stat*-p(DcOx/GDMA/DcOx)_n_, and pEtOx_50_-*stat*-pNonOx_100_-*stat*-p(DcOx/GDMA/DcOx)_n_, may be classified as amphigels. Notably, the gels of the pEtOx_100_-*stat*-pNonOx_50_ and pEtOx_50_-*stat*-pNonOx_100_ series showed very similar swelling degrees for each of the four crosslinkers, respectively. The general trends observed during the swelling in water were reproduced during swelling in ethanol: The maximum swelling degrees decreased with an increasing content of crosslinker, and the gels that contained Dc⁼Ox-derived crosslinkers showed lower swelling than the gels that contained Bu⁼Ox-derived crosslinkers. Maximum swelling was observed for pEtOx_150_-*stat*-p(BuOx/GDMA/BuOx)_6_ with a degree in the range of 6.

The swelling degrees of the 80 gels in dichloromethane (Fig. 4; see also Table SI-4 in the Supporting Information) were found to depend only on the degree of crosslinking. For the lowest degrees of crosslinking, swelling degrees in the range of 15 were obtained. Hence, all gels that did not contain any pEtOx may be classified as lipogels.

### 3.4 Polymeranalogous crosslinking and optometric determination of swelling degrees (Route 2 of Scheme 1)

In a precedent study [16], we had shown that copoly(2-oxazoline)-based lipo- and amphigels could be loaded with active pharmaceutical ingredients APIs by swelling the gels in a solution of the API in dichloromethane and subsequent drying and cleaning of the gel. As lipo- and amphigels show no significant swelling in aqueous media, drug release from such gels can only occur upon the degradation of the gel.

As additional strategy for the loading of such gels, the UV-induced polymeranalogous crosslinking was investigated in this study (Scheme 1, Route 2). The linear copoly(2-oxazoline)s were dissolved with the dithiol GDMA, the photoinitiator Lucirin TPO-L, and (eventually) ibuprofen as API in dichloromethane. These two solutions were cast into steel forms. After removal of the solvent, UV irradiation was applied in order to perform crosslinking by thiol-ene reactions. A representative disc composed of crosslinked pNonOx_150_-*stat*-pDcOx_6_ had a weight of 2.24 g with a height of 1.150 mm and a diameter of 50 mm, yielding a density of 0.99 g cm^−3^ of the gels, which is in good agreement with previously reported densities of crosslinked poly(2-oxazoline)s [30].

**Fig. 5:**
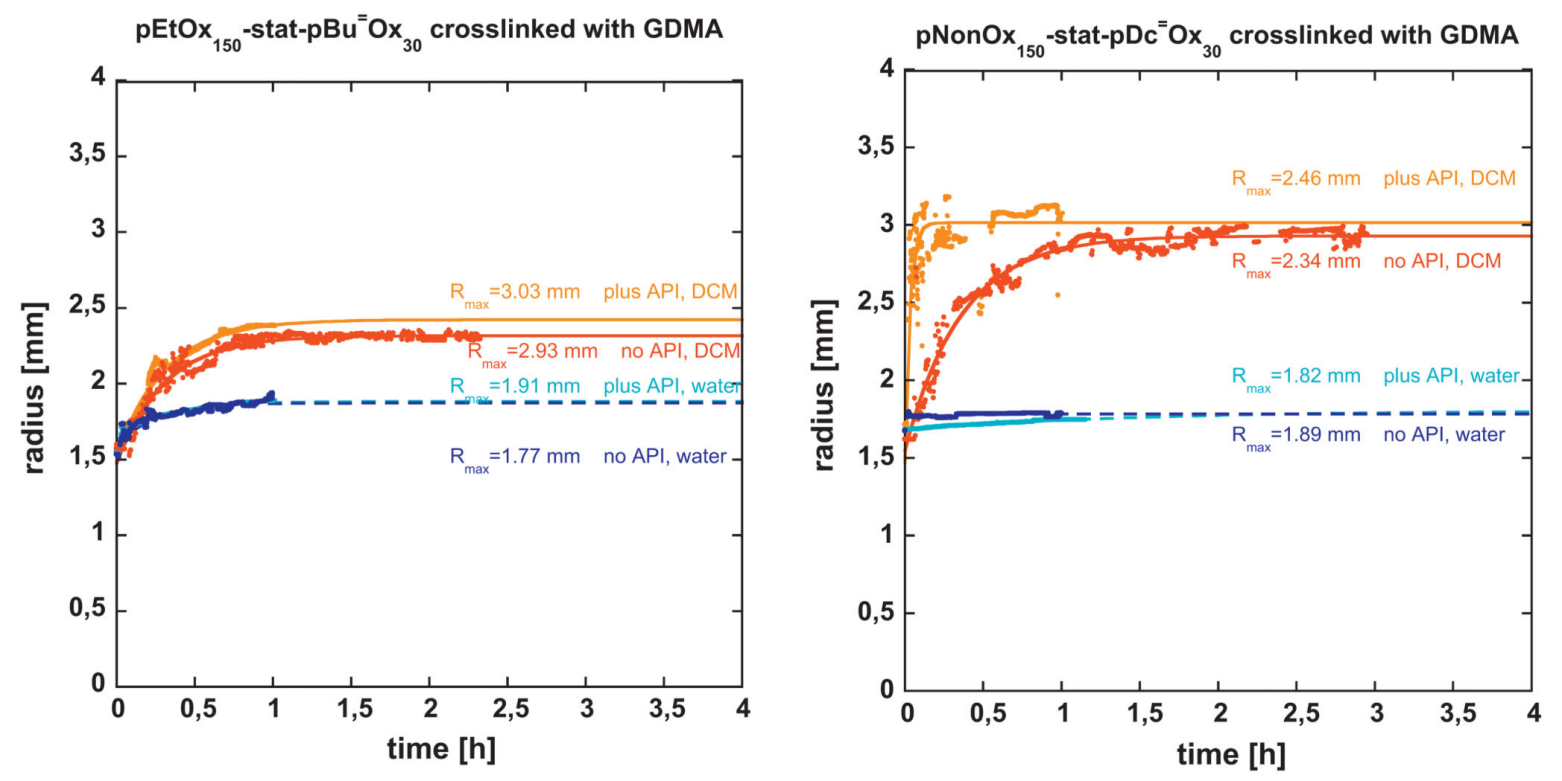
Optometrically determined radii changes during the swelling of pEtOx_150_-*stat*-pBu⁼Ox_30_ (left) and pNonOx_150_-*stat*-pDc⁼Ox_30_ (right) (both crosslinked with GDMA) in water and dichloromethane (DCM).

At room temperature, these gels were above their glass-transition temperature (Fig. 1 and Table SI-1), and small discs with a diameter of 3 mm could be punched out. These small discs were used for the optometric determination of the swelling in water and dichloromethane, aiming at a confirmation of the swelling degrees that were determined gravimetrically (Figs. 2 and 4 and Tables SI-2 and SI-4). The monitoring of the swelling properties of gels was performed on a device called FDRRT (Find Disc Radius in Real Time) and recorded with labview software. Measurements of the disc radii were performed for 1 h, the radii of the samples were plotted as a function of time (Fig. 5). The method is based on the Li and Tanaka model [31], where it has been theoretically and experimentally established that the treatment of the swelling properties of a disc gel, as a cylinder gel, is an isotropic swelling in all directions. The swelling of the gel starts immediately after the sample was immersed within the solvent. The data reported within Fig. 5 were fitted following the Tanaka model defined by Eq. (1) [31].

(1) $$\text{R}\left(\text{t}\right)={\text{R}}_{\infty }-\left({\text{R}}_{\infty }-{\text{R}}_{0}\right){\text{e}}^{\left(\ln{\text{B}}_{1}-\frac{\text{t}}{\tau_{1}}\right)},$$

in which R_∞_ and R_0_ are the radii of the disc at the equilibrium state and before swelling (R_0_ ≈ 1.5 mm), respectively, τ_1_ is the relaxation time, B_1_ is a coefficient constant, and t is the time.

From the abovementioned FDRRT data, the experimental volumetric swelling degree (SD_V_) may be obtained from Eq. (2), using the volumes V_∞_ and V_0_ of the disc at the equilibrium state and before swelling, respectively (that can be calculated from the radii R_∞_ and R_0_ as well as the heights h_∞_ and h_0_).

(2) $${\text{SD}}_{\text{V}}=\frac{{\text{V}}_{\infty }-{\text{V}}_{0}}{{\text{V}}_{0}}=\frac{{\text{V}}_{\infty }}{{\text{V}}_{0}}-1=\frac{{\text{R}}_{\infty }^{2}{\text{h}}_{\infty }}{{\text{R}}_{0}^{2}{\text{h}}_{0}}-1$$

Taking into account the isotropic swelling, constituting R_∞_/R_0_ = h_∞_/h_0_, the volumetric swelling degree may also be calculated according to Eq. (3) (cp. Table 1).

(3) $${\text{SD}}_{\text{V}}=\frac{{\text{R}}_{\infty }^{3}}{{\text{R}}_{0}^{3}}-1$$

The data of the swelling degree obtained by FDRRT have been mathematically filtered to correct the fluctuations of the gel measurement. This technique allows reconstructing plots from sets of data points to improve their quality by, for example, reducing noise and removing outliers. Of special interest was the comparison among the gravimetric and volumetric swelling degrees, the data of which have been summarized in Table 1.

Four trends become easily discernible: (i) The volume:surface ratio (powder vs. disc) does not significantly influence the maximum swelling degrees, represented by very comparable gravimetric swelling degrees for both types of specimens. (ii) The absence or presence of the API ibuprofen does not significantly alter the swelling properties, neither from the gravimetric or the volumetric point of view. (iii) Hydrogels in water as well as lipogels in dichloromethane exhibit very comparable volumetric and gravimetric swelling degrees. (iv) Lipogels in water and hydrogels in dichloromethane, however, show distinctly different volumetric and gravimetric (maximum) swelling degrees. Notably, in the case of lipogels in water, the volumetric expansion is significantly lower than the gravimetric uptake, which might be referred to the space-saving coiling of the long and flexible, hydrophobic alkyl chains. On the other hand, in the case of hydrogels in dichloromethane, the gravimetric swelling degrees are significantly larger than their volumetric analogues, which might be referred to the limited ‘stretchability’ of the short non-hydrophobic alkyl chains. These trends will be carefully monitored in a subsequent study.

### 3.5 pH-Mediated and enzymatic degradation of the polymer networks

**Fig. 6:**
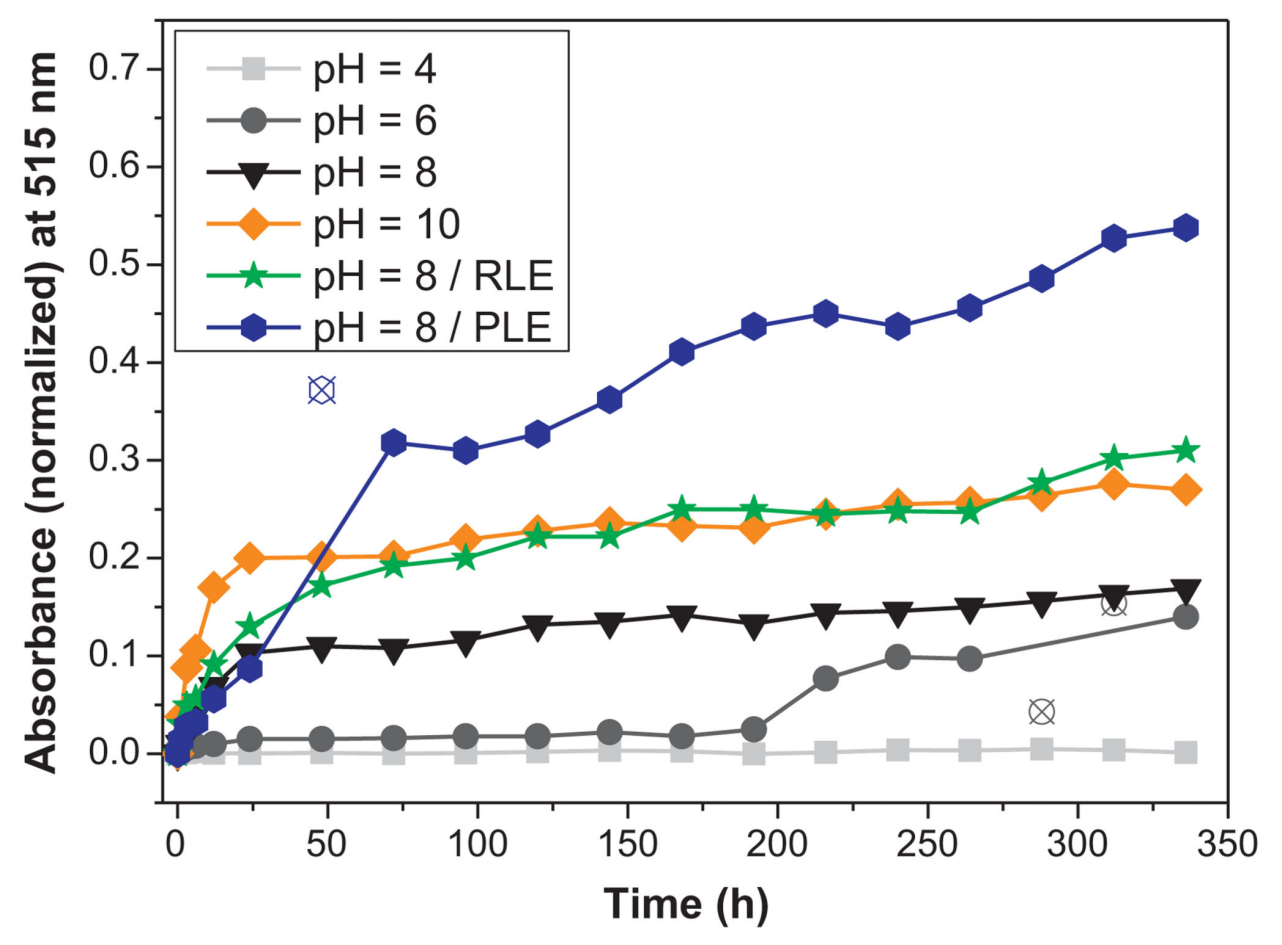
(Normalized) Absorbance at 515 nm for the quantification of the released dye Eosin B during degradation studies of crosslinked pEtOx_100_-*stat*-pNonOx_50_-*stat*-pBu⁼Ox_30_ at various conditions.

For a proof-of-concept and planning of an extended study of the degradation of the gels (that is intended to correlate the hydrophobic/hydrophilic character of the gels with the rate and order of degradation), a representative poly(2-oxazoline)-co-polyester network, namely pEtOx_100_-*stat*-pNonOx_50_-*stat*-pBu⁼Ox_30_, was dissolved with the dithiol GDMA, the photoinitiator Lucirin TPO-L, and Eosin B as UV/Vis-detectable dye in dichloromethane/ethanol. These two solutions were cast into steel forms with a diameter of 50 mm. After removal of the solvent, UV irradiation was applied in order to perform crosslinking by thiol-ene reactions. Discs of 2 mm diameter and a height of 1.25 mm were cut from these discs. Assuming the statistic distribution of the dye Eosin B, each of the small discs with a diameter of 2 mm contained 12.8 μg of the dye Eosin B. For pH-mediated as well as enzymatic degradation, sets of four discs were stored at 25 °C in 2.5 mL of aqueous solutions buffered at pH = 4, 6, 8, and 10, respectively, as well as in solutions containing esterases that were buffered at pH = 8. In case of quantitative release, a concentration of 4·12.8/2.5 = 20.5 μg mL^−1^ of Eosin B would be achieved. The degradation was monitored by UV/Vis spectroscopy for the quantification the release of the dye Eosin B for 14 days (Fig. 6).

At pH = 4, no degradation could be observed during the first two weeks; still at pH = 6, degradation was comparably slow: the absorbance of 0.14 at the end of the study corresponds to a concentration of 2 μg mL^−1^ of Eosin B, which equals 10% of the concentration achievable by quantitative release of the dye. Analogously, the rates of dye release increased at increased pH values of 8 and 10, respectively. At pH = 10, an absorbance of 0.27 was achieved within the duration of the study, which is twice the value of the absorbance achieved at pH = 6. Notably, the degradation with rabbit liver esterase (RLE) at pH = 8 was found to be very comparable to the enzyme-free degradation at pH = 10. Highest release rates were found for the porcine liver esterase (PLE)-mediated degradation at pH = 8, which yielded a solution with an absorption of 0.54 within two weeks, which is twice as high as the value obtained from degradation at pH = 10 or from the RLE-mediated degradation at pH = 8.

## 4 Conclusions and outlook

Poly(2-oxazoline)-based gels composed of monofunctional EtOx and/or NonOx (in four different ratios: pEtOx:pNonOx = 150:0, 100:50, 50:100, and 0:150) as well as one of four ether- or ester-derived difunctional 2-oxazoline monomers (five different ratios with respect to the sum of pEtOx and pNonOx: 150:6, 150:7.5, 150:10, 150:15, and 150:30) were synthesized by microwave-assisted cationic ring-opening polymerizations. The four novel crosslinkers were synthesized from the thiol-ene reactions of either GDMA or DEG (as dithiols) and Bu⁼Ox or Dc⁼Ox (as ene compounds). With respect to the targeted potential biomedical applications such as drug delivery, glass-transition of such gels should be in the range of 20–30 °C. The linear pEtOx-pNonOx copolymers had glass-transition temperatures of 56.4 ± 0.2 °C (pEtOx_150_), 20.0 ± 0.1 °C (pEtOx_100_-*stat*-pNonOx_50_), and 16.5 ± 0.1 °C (pEtOx_50_-*stat*-pNonOx_100_); pNonOx_150_ revealed no glass-transition. The compositions of the gels as well as the degrees of crosslinking, represented by the content of difunctional 2-oxazoline units, were found to alter the glass-transition temperatures. 53 of the 80 gels exhibited a glass-transition temperature in the range from −5.9 to 45.3 °C; a total of 13 derivatives exhibited glass-transition temperatures in the targeted range of 20–30 °C. Notably, for each of the four crosslinkers investigated in this study contained, at least one congener met the targeted temperature window.

Gels that did not contain any pNonOx acted as hydrogels, whereas gels that did not contain any pEtOx acted as lipogels. Gels containing pEtOx and pNonOx may be best described as amphigels. In general, an increasing degree of crosslinking, represented by the content of difunctional 2-oxazoline units, increased the network rigidity and lowered the maximum swelling degrees.

In a second route, polymeranalogous crosslinking for the preparation of crosslinked networks was investigated on the example of the UV-mediated thiol-ene reaction of pEtOx_150_-*stat*-pBu⁼Ox_30_ and GDMA. This synthetic strategy enables the preparation of specimen of defined geometry and the loading of APIs into the network in one step. The optometric determination of the swelling of the gels was found to yield swelling degrees that perfectly confirmed the findings from the gravimetric determination. The crosslinking unit GDMA contains ester bonds that can be hydrolyzed by pH stimuli as well as by esterases, and representative examples of this library of gels will be tested for drug release during enzymatic and/or pH-mediated degradation in a subsequent study, with special respect to the hydrophobic/hydrophilic character of the gels and the rate and order of degradation.

## Appendix A. Supplementary material

Supplementary data associated with this article can be found, in the online version, at http://dx.doi.org/10.1016/j.eurpolymj.2016.08.012.

SI

## Figures and Tables

**Table 1:** Gravimetrically determined swelling degrees from powders (SD_G_ powder) and disc-shaped specimens (SD_G_ disc) and the experimental volumetric swelling degrees (SD_V_) of the 8 gel/API/solvent combinations investigated (as crosslinker, GDMA was used). Also the values of the radii upon maximum swelling are listed. The gels containing API were only measured as disc-shaped specimens.

		pEtOx_150_-*stat*-pBu⁼Ox_30_ in water	pEtOx_150_-*stat*-pBu⁼Ox_30_ in dichloromethane	pNonOx_150_-*stat*-pDc⁼Ox_30_ in water	pNonOx_150_-*stat*-pDc⁼Ox_30_ in dichloromethane
Without API	R_∞_ [mm]	1.89	2.34	1.77	2.93
	SD_G_ (powder)	1.0	4.8	0.3	7.1
	SD_G_ (disc)	1.1	5.4	0.3	8.2
	SD_V_	1.0	2.8	0.6	6.45
With API	R_∞_ [mm]	1.82	2.46	1.91	3.03
	SD_G_ (disc)	1.2	6.0	0.4	8.4
	SD_V_	0.8	3.4	1.1	7.2
