# Supplementary material for: UV-mediated thiol-ene click reactions for the synthesis of drug-loadable and degradable gels based on copoly(2-oxazoline)s
Source: Eur Polym J. Author manuscript; Available in PMC 2017 Apr 1. (PMC5349497; doi:10.1016/j.eurpolymj.2016.08.012)
Supplement: SI [file NIHMS71331-supplement-SI.pdf]

UV-Mediated Thiol-Ene Click Reactions for the Synthesis of Drug-Loadable and Degradable Gels Based on Copoly(2-oxazoline)s

Klaus P. Luef, Charlotte Petit, Bettina Ottersböck, Gernot Oreski, Francis Ehrenfeld, Bruno Grassl, Stéphanie Reynaud and Frank Wiesbrock

Table SI-1: Glass-transition temperatures of the 80 copoly(2-oxazoline)-based networks.

↓ Ratio EtOx:NonOx	↓ Ratio (EtOx+NonOx):(BuOx/DEG/BuOx)				
	150:6	150:7.5	150:10	150:15	150:30
150:0	28.0 ± 4.2	23.5 ± 2.6	19.7 ± 2.8	20.0 ± 3.8	15.3 ± 2.8
100:50	24.4 ± 1.2	20.6 ± 1.3	17.3 ± 3.1	16.0 ± 1.2	9.1 ± 1.7
50:100	16.5 ± 0.2	17.0 ± 0.2	16.0 ± 0.0	10.9 ± 0.0	12.4 ± 1.2
0:150	---	---	---	---	---

↓ Ratio EtOx:NonOx	↓ Ratio (EtOx+NonOx):(BuOx/GDMA/BuOx)				
	150:6	150:7.5	150:10	150:15	150:30
150:0	23.2 ± 1.4	25.2 ± 0.5	19.1 ± 1.7	15.8 ± 3.5	2.3 ± 0.7
100:50	24.6 ± 0.9	20.8 ± 2.1	19.9 ± 1.0	18.6 ± 1.4	18.6 ± 3.3
50:100	17.9 ± 0.5	17.6 ± 0.5	14.9 ± 0.5	12.6 ± 0.9	11.6 ± 1.1
0:150	---	---	---	---	11.1 ± 1.2

↓ Ratio EtOx:NonOx	↓ Ratio (EtOx+NonOx):(DcOx/DEG/DcOx)				
	150:6	150:7.5	150:10	150:15	150:30
150:0	45.3 ± 1.4	31.2 ± 3.1	27.8 ± 3.1	21.4 ± 2.1	8.8 ± 0.7
100:50	18.7 ± 1.2	15.7 ± 1.0	15.2 ± 0.5	8.0 ± 0.9	–5.9 ± 0.2
50:100	---	---	---	---	0.8 ± 4.9
0:150	---	---	---	---	---

↓ Ratio EtOx:NonOx	↓ Ratio (EtOx+NonOx):(DcOx/GDMA/DcOx)				
	150:6	150:7.5	150:10	150:15	150:30
150:0	27.1 ± 5.0	40.3 ± 1.7	18.9 ± 1.9	13.4 ± 0.2	7.2 ± 0.5
100:50	14.1 ± 1.7	16.0 ± 1.2	11.1 ± 0.7	7.2 ± 1.4	–1.0 ± 0.5
50:100	---	---	---	---	–2.6 ± 0.2
0:150	---	---	---	---	---

Table SI-2: Swelling degrees of the 80 copoly(2-oxazoline)-based networks in water.

↓ Ratio EtOx:NonOx	↓ Ratio (EtOx+NonOx):(BuOx/DEG/BuOx)				
	150:6	150:7.5	150:10	150:15	150:30
150:0	6.0 ± 0.2	4.1 ± 0.2	2.9 ± 0.9	2.3 ± 0.1	1.3 ± 0.1
100:50	1.3 ± 0.1	0.7 ± 0.1	0.7 ± 0.0	0.6 ± 0.1	0.8 ± 0.2
50:100	0.5 ± 0.1	0.4 ± 0.0	0.7 ± 0.2	0.5 ± 0.0	0.6 ± 0.1
0:150	0.4 ± 0.1	0.5 ± 0.1	0.4 ± 0.0	0.4 ± 0.0	0.6 ± 0.1

↓ Ratio EtOx:NonOx	↓ Ratio (EtOx+NonOx):(BuOx/GDMA/BuOx)				
	150:6	150:7.5	150:10	150:15	150:30
150:0	6.5 ± 0.4	3.9 ± 0.6	3.2 ± 0.2	2.4 ± 0.5	0.8 ± 0.0
100:50	1.0 ± 0.2	0.7 ± 0.0	0.6 ± 0.0	0.6 ± 0.0	1.0 ± 0.0
50:100	0.5 ± 0.1	0.5 ± 0.0	0.4 ± 0.0	1.0 ± 0.2	0.5 ± 0.1
0:150	0.5 ± 0.1	0.5 ± 0.0	0.5 ± 0.0	0.5 ± 0.1	0.5 ± 0.1

↓ Ratio EtOx:NonOx	↓ Ratio (EtOx+NonOx):(DcOx/DEG/DcOx)				
	150:6	150:7.5	150:10	150:15	150:30
150:0	2.6 ± 0.1	2.0 ± 0.1	1.8 ± 0.3	1.0 ± 0.1	0.7 ± 0.1
100:50	0.9 ± 0.1	1.0 ± 0.1	0.7 ± 0.2	0.4 ± 0.0	0.5 ± 0.1
50:100	0.4 ± 0.1	0.4 ± 0.1	0.7 ± 0.1	0.7 ± 0.1	0.6 ± 0.0
0:150	0.3 ± 0.0	0.5 ± 0.0	0.5 ± 0.0	0.5 ± 0.1	0.5 ± 0.3

↓ Ratio EtOx:NonOx	↓ Ratio (EtOx+NonOx):(DcOx/GDMA/DcOx)				
	150:6	150:7.5	150:10	150:15	150:30
150:0	3.5 ± 0.3	2.0 ± 0.0	1.9 ± 0.3	1.2 ± 0.2	1.0 ± 0.2
100:50	1.1 ± 0.5	0.8 ± 0.1	0.8 ± 0.1	0.5 ± 0.0	0.8 ± 0.2
50:100	0.4 ± 0.1	0.3 ± 0.1	0.4 ± 0.0	0.5 ± 0.0	0.7 ± 0.1
0:150	0.4 ± 0.1	0.3 ± 0.0	0.4 ± 0.1	0.4 ± 0.0	0.3 ± 0.1

Table SI-3: Swelling degrees of the 80 copoly(2-oxazoline)-based networks in ethanol.

↓ Ratio EtOx:NonOx	↓ Ratio (EtOx+NonOx):(BuOx/DEG/BuOx)				
	150:6	150:7.5	150:10	150:15	150:30
150:0	5.4 ± 0.0	4.1 ± 0.4	3.1 ± 0.2	2.4 ± 0.1	1.3 ± 0.0
100:50	5.4 ±0.0	4.4 ± 0.3	3.9 ± 0.3	2.6 ± 0.0	1.5 ± 0.0
50:100	4.8 ± 0.0	3.8 ± 0.0	3.3 ± 0.1	2.4 ± 0.1	1.4 ± 0.0
0:150	1.7 ± 0.0	1.1 ± 0.0	0.6 ± 0.0	0.5 ± 0.0	0.5 ± 0.0

↓ Ratio EtOx:NonOx	↓ Ratio (EtOx+NonOx):(BuOx/GDMA/BuOx)				
	150:6	150:7.5	150:10	150:15	150:30
150:0	6.3 ± 0.0	5.2 ± 0.3	3.6 ± 0.4	2.5 ± 0.3	1.4 ± 0.2
100:50	6.0 ± 0.2	5.0 ± 0.0	3.6 ± 0.1	3.0 ± 0.1	1.9 ± 0.1
50:100	4.9 ± 0.1	4.3 ± 0.4	3.7 ± 0.1	2.7 ± 0.0	1.7 ± 0.0
0:150	0.6 ± 0.2	0.5 ± 0.0	0.6 ± 0.0	0.9 ± 0.1	1.2 ± 0.1

↓ Ratio EtOx:NonOx	↓ Ratio (EtOx+NonOx):(DcOx/DEG/DcOx)				
	150:6	150:7.5	150:10	150:15	150:30
150:0	4.4 ± 0.2	3.8 ± 0.2	3.2 ± 0.3	2.3 ± 0.1	1.5 ± 0.1
100:50	4.4 ± 0.1	3.9 ± 0.2	3.4 ± 0.0	2.3 ± 0.2	1.3 ± 0.1
50:100	3.3 ± 0.0	2.6 ± 0.1	2.5 ± 0.0	1.7 ± 0.0	1.5 ± 0.1
0:150	0.4 ± 0.0	0.5 ± 0.0	0.7 ± 0.1	1.0 ± 0.0	1.2 ± 0.1

↓ Ratio EtOx:NonOx	↓ Ratio (EtOx+NonOx):(DcOx/GDMA/DcOx)				
	150:6	150:7.5	150:10	150:15	150:30
150:0	5.3 ± 0.1	3.5 ± 0.0	3.3 ± 0.1	2.4 ± 0.1	1.3 ± 0.1
100:50	3.9 ± 0.1	3.8 ± 0.2	3.2 ± 0.1	2.3 ± 0.1	1.4 ± 0.0
50:100	3.8 ± 0.2	3.7 ± 0.3	3.2 ± 0.1	2.1 ± 0.0	1.2 ± 0.0
0:150	0.4 ± 0.0	0.5 ± 0.0	0.5 ± 0.0	0.8 ± 0.0	1.1 ± 0.0

Table SI-4: Swelling degrees of the 80 copoly(2-oxazoline)-based networks in dichloromethane.

↓ Ratio EtOx:NonOx	↓ Ratio (EtOx+NonOx):(BuOx/DEG/BuOx)				
	150:6	150:7.5	150:10	150:15	150:30
150:0	13.2 ± 3.4	9.6 ± 0.6	8.2 ± 0.5	6.2 ± 0.7	4.4 ± 0.4
100:50	13.7 ± 1.1	11.9 ± 0.0	8.0 ± 0.8	7.5 ± 0.9	5.2 ± 0.6
50:100	14.0 ± 0.6	12.5 ± 0.7	10.1 ± 0.9	8.0 ± 0.8	5.0 ± 0.4
0:150	13.2 ± 1.4	11.4 ± 1.6	10.2 ± 1.1	8.5 ± 0.8	7.1 ± 0.8

↓ Ratio EtOx:NonOx	↓ Ratio (EtOx+NonOx):(BuOx/GDMA/BuOx)				
	150:6	150:7.5	150:10	150:15	150:30
150:0	15.2 ± 0.6	12.3 ± 1.4	8.6 ± 1.0	6.8 ± 0.8	4.8 ± 0.6
100:50	15.7 ± 0.2	13.6 ± 1.1	10.6 ± 1.2	9.7 ± 1.2	6.5 ± 0.7
50:100	16.6 ± 0.0	14.3 ± 0.5	13.1 ± 0.9	10.4 ± 0.8	6.0 ± 0.6
0:150	17.8 ± 0.9	15.1 ± 1.1	13.1 ± 4.2	10.9 ± 0.5	6.2 ± 0.8

↓ Ratio EtOx:NonOx	↓ Ratio (EtOx+NonOx):(DcOx/DEG/DcOx)				
	150:6	150:7.5	150:10	150:15	150:30
150:0	10.1 ± 0.6	10.2 ± 1.3	7.6 ± 1.1	7.1 ± 1.1	5.4 ± 1.0
100:50	11.8 ± 0.0	11.8 ± 0.8	8.7 ± 1.0	7.0 ± 1.2	5.3 ± 0.8
50:100	12.8 ± 0.4	11.2 ± 0.3	9.2 ± 1.0	8.0 ± 1.0	5.9 ± 0.5
0:150	11.7 ± 1.4	11.1 ± 1.1	8.7 ± 0.5	7.6 ± 1.1	5.6 ± 0.8

↓ Ratio EtOx:NonOx	↓ Ratio (EtOx+NonOx):(DcOx/GDMA/DcOx)				
	150:6	150:7.5	150:10	150:15	150:30
150:0	13.5 ± 1.0	9.6 ± 0.9	8.9 ± 0.9	7.8 ± 1.8	5.4 ± 0.5
100:50	13.3 ± 1.7	10.7 ± 0.8	11.0 ± 1.5	9.1 ± 1.5	5.5 ± 0.6
50:100	11.0 ± 1.2	13.8 ± 0.9	15.1 ± 1.2	8.6 ± 1.1	6.3 ± 0.7
0:150	14.4 ± 0.6	9.9 ± 0.9	9.4 ± 1.5	7.3 ± 1.2	7.1 ± 1.0
